# Prevalence of anemia and associated factors among indigenous children in Brazil: results from the First National Survey of Indigenous People’s Health and Nutrition

**DOI:** 10.1186/1475-2891-12-69

**Published:** 2013-05-28

**Authors:** Maurício S Leite, Andrey M Cardoso, Carlos EA Coimbra, James R Welch, Silvia A Gugelmin, Pedro Cabral I Lira, Bernardo L Horta, Ricardo Ventura Santos, Ana Lúcia Escobar

**Affiliations:** 1 Departamento de Nutrição, Centro de Ciências da Saúde, Universidade Federal de Santa Catarina, Florianópolis, SC 88040-900, Brazil; 2 Escola Nacional de Saúde Pública, Fundação Oswaldo Cruz, Rua Leopoldo Bulhões 1480, Rio de Janeiro, RJ 21041-210, Brazil; 3 Instituto de Saúde Coletiva, Universidade Federal de Mato Grosso, Av. Fernando Correa da Costa 2367, Cuiabá, MT 78060-900, Brazil; 4 Departamento de Nutrição, Universidade Federal de Pernambuco, Avenida Professor Moraes Rego 1235, Recife, PE 50670-901, Brazil; 5 Programa de Pós-Graduação em Epidemiologia, Universidade Federal de Pelotas, Rua Marechal Deodoro 1160, Pelotas, RS 96020-220, Brazil; 6 Departamento de Antropologia, Museu Nacional, Universidade Federal do Rio de Janeiro, Quinta da Boa Vista s/n, Rio de Janeiro, RJ 20940-040, Brazil; 7 Departamento de Ciências Biomédicas, Universidade Federal de Rondônia, Rodovia BR-364 Km 9.5, Porto Velho, RO 76801-059, Brasil

**Keywords:** Brazil, Indigenous peoples, Health surveys, Nutrition surveys, Health status indicators, Anemia, Child health

## Abstract

**Background:**

Anemia is the most prevalent nutritional deficiency globally, affecting about a quarter of the world population. In Brazil, about one-fifth of children under five years of age are anemic. Previous case studies indicate prevalence rates much higher among indigenous peoples in the country. The First National Survey of Indigenous People’s Health and Nutrition in Brazil, conducted in 2008–2009, was the first survey based on a nationwide representative sample to study the prevalence of anemia and associated factors among indigenous children in Brazil.

**Methods:**

The survey assessed the health and nutritional status of indigenous children < 5 years of age based on a representative sample of major Brazilian geopolitical regions. A stratified probabilistic sampling was carried out for indigenous villages. Within villages, children < 5 years of age in sampled households were included in the study. Prevalence rates of anemia were calculated for independent variables and hierarchical multivariate analysis were conducted to assess associations.

**Results:**

Evaluation of hemoglobin levels was conducted for 5,397 children (88.1% of the total sample). The overall prevalence of anemia was 51.2%. Higher risk of presenting anemia was documented for boys, lower maternal schooling, lower household socioeconomic status, poorer sanitary conditions, presence of maternal anemia, and anthropometric deficits. Regional differences were observed, with the highest rate being observed in the North.

**Conclusions:**

The prevalence rates of anemia in indigenous children were approximately double than those reported for non-indigenous Brazilian children in the same age group. Similarly notable differences in the occurrence of anemia in indigenous and non-indigenous children have been reported for other countries. Deeper knowledge about the etiology of anemia in indigenous children in Brazil is essential to its proper treatment and prevention.

## Introduction

Anemia is considered the most prevalent nutritional deficiency globally, affecting about a quarter of the world population, especially children and women of reproductive age [1,2]. In children, anemia can negatively affect cognitive development, school performance, physical growth, and immunity [3-6]. In Brazil, the changing nutritional scenario of children less than five years of age has been evaluated optimistically based on results from national surveys in recent decades, revealing a tendency towards dramatic reductions in the prevalence rates of chronic undernutrition for this age group in all regions of the country [7]. This trend has been attributed to improvements in education levels and family socioeconomic conditions, as well as public investments in infrastructure, health, and sanitation. Nevertheless, similar improvements have not been observed in relation to child anemia in Brazil. According to recent studies, about one-fifth of Brazilian children under five years of age are anemic [8,9].

The few existing studies of anemia among indigenous peoples in Brazil, which are derived from research conducted in specific localized groups, have revealed prevalence rates much higher than those documented for the national population, generally greater than 50-60% in children under five years of age [10-12]. The discrepancy observed between the prevalence rates of anemia among indigenous and non-indigenous children in Brazil are also documented for other regions of the world, with the burden of disease tending to be much higher among indigenous peoples than their respective national populations [13-16].

Until now there were no population-based studies permitting generalization about the epidemiology of anemia and its principal determinants among indigenous children in Brazil. The First National Survey of Indigenous People’s Health and Nutrition in Brazil (henceforth, “National Survey”), conducted in 2008–2009, was the first study of its kind to include a nationwide representative sample of indigenous peoples in the country [17]. One of its main objectives was to understand the contemporary health profile of the nation’s indigenous population in terms of diverse socioeconomic and environmental factors. The aim of our paper is to present the results of analyses on the prevalence of anemia and associated factors among indigenous children ≥ 6 months and < 5 years of age in Brazil, based on hemoglobin levels measured by the National Survey field teams.

## Methods

The National Survey assessed the health and nutritional status of indigenous children < 5 years of age in Brazil [17]. A representative sample of four major Brazilian geopolitical regions (North, Northeast, Central-West and Southeast/South) was obtained by multi-stage sampling, as previously reported [17]. Initially, a stratified probabilistic sampling was carried out of indigenous villages located in the four regions, based on a list of villages in federal indigenous reserves provided by the Brazilian Ministry of Health’s National Health Foundation (Fundação Nacional de Saúde – FUNASA) on January 22, 2008. The original list contained 3,995 villages. Excluded for the sampling purposes of the National Survey were 1227 (30.7%) villages identified by FUNASA as vacated (“desaldeadas”), deactivated, or having less than 31 inhabitants. The sample size for each region was estimated based on the size of its target population, a prevalence of 50% for all outcomes, a relative precision of 5%, and a confidence level of 95%, according to the methodology proposed by Lemeshow et al. [18]. Taking into account the calculated sample size for each region, Sequential Poisson Sampling criteria were used to select villages for inclusion in the study [19]. The final sample included 123 villages distributed by region as follows: 65 (North), 14 (Central-West), 23 (Northeast), and 21 (South/Southeast).

Subsequently, indigenous households in sampled villages were selected by either census or sample, depending on their estimated populations. Investigation by census (inclusion of all households) was carried out in villages with populations of children < 5 years and women from 14 to 49 years of age ≤ 150 individuals. In villages with populations of women and children greater than 150 individuals, households were selected systematically using a predetermined increment without substitution in the event of absence or refusal. Further details on the study methodology have been published elsewhere [17].

In investigated households, the mothers or caretakers of all children < 5 years of age were interviewed. The instrument contained questions on infant feeding, access to health services, morbidity and nutritional status, the latter of which was clinically evaluated by our teams using anthropometry and hemoglobin, as well as household characteristics [17,20]. Sources of household income were classified as “regular” if received monthly or annually (e.g., salaries, pensions, social benefits). Basic birth data were obtained from FUNASA healthcare records, personal documents, or informed by interviewees.

In order to determine hemoglobin levels in infants and children ≥ 6 months and < 5 years of age*,* a single drop of capillary blood was obtained with one-way lancets fitted to an ACCU-CHEK® lancing device by Roche (Mannheim, Germany) and analyzed using portable hemoglobinometers, model HemoCue Hb 201+ (Ängelholm, Sweden). Children with hemoglobin levels < 11.0 g/dL were considered anemic [3]. Levels < 9.5 g/dL were considered indicative of moderate/severe anemia [3].

Principal component analysis of reported quantities of 19 durable goods was used to calculate an indicator of socioeconomic condition [17]. Factor scores from the first component, accounting for 19% of the total variability in the dataset (eigenvalue of 3.56), were applied as weights to the item quantities reported for each household. The sum of these values was then used to classify households in tertiles, based on the combined distribution and considering the four regions, which served as a household goods index.

In initial data analyses, prevalence rates of anemia were calculated according to independent variables (geopolitical region, demographic variables, socioeconomic variables, characteristics of the household environment, and maternal and child characteristics). Estimates were corrected for the complex sampling design of the study. Measures of associations were expressed as prevalence ratios and their corresponding 95% confidence intervals. Chi-square tests for linear trend were used to evaluate differences in proportions. The prevalence of moderate/severe anemia was calculated by geopolitical region, even though this outcome was not included separately in the other analyses.

The multivariate analysis followed a hierarchical conceptual framework based on Poisson regression with robust adjustment of the variance for dichotomous outcomes (anemic or not anemic) [21,22]. Geopolitical region was used as a control variable in all levels. Other independent variables entered the analysis at their respective levels in the model. The first and most distal level included child’s sex, age and socioeconomic variables (maternal schooling, presence of regular income from salaries or social programs, and household goods index). The second hierarchical level contained variables related to household construction (flooring, walls, and roofing), composition (number of residents and number of children < 5 years in the household), electricity, and sanitation (location used to defecate, predominant destination of household trash, and source of drinking water). The third level included maternal characteristics (age, number of children ever had, and anemia). Finally, the fourth and most proximal level encompassed child health variables (place of delivery, birth weight, nutritional status, diarrhea during the prior week, and treatment or supplementation with iron sulfate in the prior three months).

The initial step of the multivariate analyses was to select variables with p < 0.20 following adjustment for geopolitical region and for variables retained in previous levels, if present. Subsequently, for each level, selected variables were jointly included in the model, controlling for geopolitical region and all variables retained in previous levels, if present. A backward procedure was then used to progressively exclude variables at each level, retaining only those with a significance level of p < 0.05. Therefore, in the final model, the prevalence ratios were simultaneously adjusted for variables in the same hierarchical level, those retained in previous levels, and geopolitical region. All analyses were conducted with the program STATA 10.0 (College Station, TX, USA).

### Ethics

The National Survey was authorized by the National Committee on Research Ethics (Comissão Nacional de Ética em Pesquisa – CONEP, authorization number 256/2008) and the National Indian Foundation (Fundação Nacional do Índio – FUNAI). Upon arrival at each indigenous community, the research team held meetings with leaders and other community members, during which the objectives and procedures of the study were clearly presented. A Free and Informed Collective Consent form was presented and signed by leaders, as well as other community representatives when indicated to our field teams (indigenous health agents, teachers, etc.). Any particular village, household, parent, or guardian was allowed to decline to participate at any moment of fieldwork.

## Results

Evaluation of hemoglobin levels was conducted for 5,397 children (88.1% of the planned sample) in 113 villages (91.9% of sample) and 5,305 households (93.5% of sample). The overall prevalence of anemia (Hb < 11 g/dL) was 51.2%. Among all geopolitical regions, the highest prevalence was recorded in the North (66.4%), followed by the Central-West (51.5%), South/Southeast (48.0%) and Northeast (41.1%) (Table 1). The prevalence of moderate/severe anemia (Hb < 9 g/dL) was 16.4%, distributed by region in the same order as described for anemia: North (25.4%), Central-West (14.8%), South/Southeast (15.9%) and Northeast (10.1%).

**Table 1:** Prevalence rates of anemia among indigenous children < 60 months according to geopolitical region, sex, age, and socioeconomic characteristics, First National Survey of Indigenous People’s Health and Nutrition, Brazil, 2008–2009

**Characteristic studied**	**N**	**Prevalence (%)**	**PR**	**CI 95%**
**Region**				
Northeast	1211	41.06	1.00	Reference
South/Southeast	765	47.95	1.17	0.95-1.44
Central-West	1141	51.52	1.25	1.04-1.51
North	2280	66.40	1.62	1.36-1.92
**Child's sex**^†^				
Female	2635	49.60	1.00	Reference
Male	2761	52.79	1.06	1.01-1.13
**Child’s age (months)**^†^			p = 0.000*	
6–11	673	80.24	1.00	Reference
12–23	1198	68.21	0.85	0.78-0.92
24–35	1170	48.78	0.61	0.55-0.67
36–47	1239	39.42	0.49	0.44-0.55
48–59	1117	32.86	0.41	0.36-0.47
**Maternal schooling (years)**^†^			p = 0.000*	
0	950	57.23	1.39	1.23-1.56
1–4	2370	52.70	1.28	1.15-1.42
5–9	1345	49.90	1.21	1.08-1.35
≥ 10	683	41.25	1.00	Reference
**Regular income**^†^				
Yes	2217	48.97	1.00	Reference
No	3168	52.86	1.08	1.01-1.15
**Household goods index (tertile)**^†^			p = 0.000*	
1st	1802	59.85	1.38	1.23-1.54
2nd	2083	51.73	1.19	1.09-1.30
3rd	1512	43.50	1.00	Reference

*PR* Prevalence ratio.

*CI* Confidence interval.

*χ^2^ test for linear trend.

^†^Variable retained for multivariate analysis.

Boys in all age groups had a slightly higher risk of presenting anemia than girls (PR 1.06, CI_95%_ 1.01-1.13) (Table 1). The risk of anemia decreased with increasing age of the child, progressively lowering with each age group. Maternal schooling showed a similar pattern, with lower risk among children whose mothers had ten or more years of schooling. Access to regular income and household goods index in the upper two tertiles also showed a protective effect. Belonging to the first and third tertiles of the household goods index showed the most pronounced contrast, with the former resulting in 1.38 times greater risk (CI_95%_ 1.23-1.54) than the latter.

With regard to household and environmental variables (Table 2), the risk of presenting anemia was higher in households with floors made of wood, walls of palm thatch, and roofing of nondurable materials such as canvas, and plastic. Higher risk of anemia was also observed among children in households with discontinuous electricity and drinking water originating from rivers, lakes, or open reservoirs. It was also higher in households with the predominant defecation location being outdoors in the open and in those with trash disposal by means of discarding in a river, lake, or ocean. Considering the variables related to household composition, children were more prone to have anemia if they lived in households with nine or more total residents and four or more children less than 5 years of age.

**Table 2:** Prevalence rates of anemia among indigenous children < 60 months according to physical, demographic, and sanitation characteristics of the household, First National Survey of Indigenous People’s Health and Nutrition, Brazil, 2008–2009

**Characteristic studied**	**N**	**Prevalence (%)**	**PR**	**CI 95%**
**Type of flooring**^†^				
Ceramic	462	38.95	1.00	Reference
Cement	1578	44.70	1.15	0.99-1.34
Wood	1405	60.83	1.56	1.29-1.89
Dirt	1922	56.55	1.45	1.23-1.72
**Type of walls**^†^				
Brick	1863	42.74	1.00	Reference
Mud	764	54.44	1.27	1.12-1.45
Wood	1966	60.46	1.41	1.24-1.62
Thatch	297	64.71	1.51	1.28-1.79
Canvas, plastic, or other	498	49.54	1.16	1.00-1.34
**Type of roofing**^†^				
Clay tile	1558	43.60	1.00	Reference
Corrugated sheets	2054	52.34	1.20	1.04-1.39
Wood or thatch	1735	60.87	1.40	1.22-1.59
Canvas, plastic, or other	42	66.34	1.52	1.18-1.96
**Electricity in the home**				
Yes	3129	47.56	1.00	Reference
Yes, but discontinuous	841	59.70	1.26	1.10.-1.43
No	1414	59.14	1.24	1.12-1.38
**No. of residents in household**^†^			p = 0.000*	
≤ 4	1273	46.01	1.00	Reference
5–8	2641	50.84	1.10	1.02-1.20
≥ 9	1464	58.63	1.27	1.14-1.42
**No. of children < 5 years old in household**^†^			p = 0.000*	
1	1885	47.63	1.00	Reference
2	2269	51.33	1.08	0.98-1.18
3	868	56.95	1.20	1.08-1.32
≥ 4	355	61.67	1.29	1.12-1.49
**Defecation location**				
Indoor household facility	768	41.26	1.00	Reference
Outdoor household facility	2719	53.33	1.29	1.10-1.51
Outdoors in the open	1865	54.07	1.31	1.12-1.54
Other	26	34.10	0.83	0.51-1.35
**Predominant destination of the household trash**				
Collected by removal service	640	44.00	1.00	Reference
Buried, discarded, or burned in the village	4378	52.22	1.19	1.02-1.38
Buried, discarded, or burned outside the village	305	58.17	1.32	1.03-1.70
Discarded in a river, lake, or ocean	54	70.91	1.61	1.25-2.08
Other	10	51.72	1.18	0.68-2.04
**Source of drinking water**^†^				
Faucet inside house	804	45.87	1.00	Reference
Faucet outside house	2639	51.78	1.13	1.00-1.28
Shallow well	436	50.23	1.09	0.91-1.32
River, lake, or reservoir	744	61.55	1.34	1.13-1.59
Other	766	51.17	1.12	0.86-1.45

*PR* Prevalence ratio.

*CI* Confidence interval.

*χ^2^ test for linear trend.

^†^Variable retained for multivariate analysis.

Among the maternal variables in level 3 (Table 3), maternal anemia and age were associated with the prevalence rates of child anemia, while number of children ever had was not. Children whose mothers were anemic presented a prevalence of anemia 1.35 times greater than those whose mothers had normal hemoglobin levels (CI_95%_ 1.26-1.44). Maternal age showed a protective effect for anemia in children, with prevalence ratio between the first (less than 20 years of age) and the last stratum (40 years and over) reaching 0.75 (CI_95%_ 0.65-0.86).

**Table 3:** Prevalence rates of anemia among indigenous children < 60 months according to maternal characteristics, First National Survey of Indigenous People’s Health and Nutrition, Brazil, 2008–2009

**Characteristic studied**	**N**	**Prevalence (%)**	**PR**	**CI 95%**
**Maternal age (years)**^†^			p = 0.000*	
< 20	655	61.33	1.00	Reference
20–29	2759	50.25	0.82	0.75-0.89
30–39	1541	50.17	0.82	0.74-0.91
≥ 40	428	46.03	0.75	0.65-0.86
**Number of children ever had**			p = 0.829*	
0–1	775	53.68	1.00	Reference
2	979	48.71	0.91	0.81-1.01
3	771	47.66	0.89	0.78-1.01
≥ 4	2821	52.38	0.98	0.87-1.09
**Maternal anemia**^†^				
No	3287	45.62	1.00	Reference
Yes	2010	61.65	1.35	1.26-1.44

*PR* Prevalence ratio.

*CI* Confidence interval.

*χ^2^ test for linear trend.

^†^Variable retained for multivariate analysis.

Of the child characteristics presented in Table 4, birth weight did not significantly affect the occurrence of anemia, but birth in a village as opposed to in hospitals or other locations outside the village resulted in higher risk of anemia diagnosis (PR 1.23; CI_95%_ 1.12-1.36). With regard to indicators of nutritional status, anthropometric deficits measured in terms of weight-for-age, height-for-age, and weight-for-height increased the prevalence of anemia (Table 4). Reports of diarrhea during the prior week also resulted in significantly greater prevalence of anemia (PR 1.32, CI_95%_ 1.21-1.43). Children who were reported to have received iron sulfate treatment or supplementation during the prior three months represented just 15.4% of the total sample and did not present lower prevalence rates of anemia than other children (PR 1.02, CI_95%_ 0.90-1.15).

**Table 4:** Prevalence rates of anemia among indigenous children < 60 months according to place of delivery, nutritional status, occurrence of diarrhea, and use of iron sulfate, First National Survey of Indigenous People’s Health and Nutrition, Brazil, 2008–2009

**Characteristic studied**	**N**	**Prevalence (%)**	**PR**	**CI 95%**
**Place of delivery**^†^				
Hospital	3235	47.88	1.00	Reference
Village	2141	58.96	1.23	1.12-1.36
**Birth weight (grams)**				
≥ 2500	3202	50.82	1.00	Reference
< 2500	271	50.83	1.00	0.86-1.17
**Weight-for-age (z-score)**^†^			p = 0.000*	
≥ 0	1502	46.07	1.00	Reference
< 0 and ≥ −1	2135	49.29	1.07	0.98-1.16
< −1	1749	60.28	1.31	1.21-1.41
**Height-for-age (z-score)**^†^			p = 0.000*	
≥ 0	602	41.00	1.00	Reference
< 0 and ≥ −1	1210	46.00	1.12	1.01-1.25
< −1	3533	55.83	1.36	1.21-1.53
**Weight-for-height (z-score)**^†^			p = 0.212*	
≥ 0	3471	50.84	1.00	Reference
< 0 and ≥ −1	1508	51.12	1.00	0.93-1.09
< −1	369	57.16	1.12	1.01-1.26
**Diarrhea during the prior week**^†^				
No	3847	47.58	1.00	Reference
Yes	1509	63.02	1.32	1.22-1.43
**Iron sulfate use**				
Yes	893	50.50	1.00	Reference
No	4388	51.30	1.02	0.90-1.15

*PR* Prevalence ratio.

*CI* Confidence interval.

*χ^2^ test for linear trend.

^†^Variable retained for multivariate analysis.

**Figure 1:**
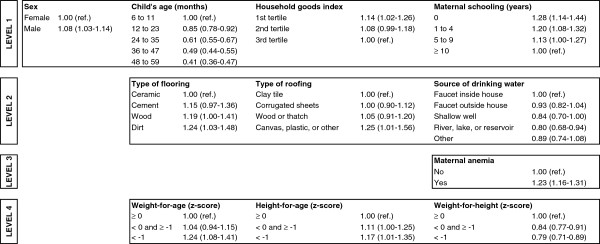
**Hierarchical model for anemia among children < 5 years of age, First National Survey of Indigenous People’s Health and Nutrition, Brazil, 2008–2009.** Obs: Values represent prevalence ratios with confidence intervals in parentheses. Geopolitical region was included as a control variable at all levels.

Remaining in the final hierarchical model for anemia were the following variables: geopolitical region, child’s sex and age, household goods index, maternal schooling, type of household flooring and roofing, source of drinking water, maternal anemia, and all anthropometric indices (Figure 1). All variables showed patterns of association consistent with those observed in the univariate analyses with the exceptions of source of drinking water and weight-for-height. In these cases, household drinking water from rivers, lakes, or reservoirs and lower weight-for-height showed protective effects for anemia.

## Discussion and conclusion

The results of the National Survey reveal that more than half of indigenous children in Brazil presented anemia (51.2%) and approximately one-sixth presented moderate/severe anemia (16.4%). These results are similar in scale to those from the few existing epidemiological studies of anemia among indigenous children in Brazil, which highlighted the significance of this nutritional deficit in the epidemiological profile of members of localized communities, ethnic groups, and populations [10-12].

According to the results of the National Survey, the prevalence rates of anemia in indigenous children nationally were approximately double those reported for non-indigenous Brazilian children in the same age group. According to the first nationwide survey assessing the occurrence of anemia in children < 5 years of age in Brazil, the National Survey on Demography and Health of Women and Children (Pesquisa Nacional de Demografia e Saúde da Criança e da Mulher – PNDS), which did not systematically include indigenous populations, the reported prevalence rates for anemia and moderate/severe anemia were 20.9% and 8.7%, respectively [8]. The pattern of inequality in the occurrence of anemia in indigenous and non-indigenous children in Brazil is also quite pronounced when comparing the frequencies observed among the four geopolitical regions of the country studied in the National Survey. The prevalence ratios of anemia among indigenous and non-indigenous children < 5 years were 1.6 in the Northeast, 4.7 in the Central-West, and 6.4 in the North. A prevalence ratio could not be calculated for the South/Southeast region because, whereas we combined the prevalence of anemia for these regions (48.0%), the PNDS presented them separately for the South (21.5%) and Southeast (22.6%) regions [8].

The geographical distribution of anemia in indigenous children reveals prevalence rates to be highest in the North (66.4%), followed by the Central-West (51.5%), South/Southeast (48.0%), and Northeast (41.1%). This notable regional difference follows a similar pattern to that observed in the National Survey for other nutritional measures, such as the prevalence of chronic undernutrition among indigenous children [20]. Additionally, indigenous households in the North region were observed to have lower sanitation, maternal schooling, and socioeconomic indicators as compared to all other regions [17]. These findings suggest that nationwide generalizations about indicators of nutritional status in indigenous children in Brazil, such as anemia and undernutrition, can conceal important regional patterns.

The notable difference in the occurrence of anemia in indigenous and non-indigenous children in Brazil documented by the National Survey finds parallel in results from other countries. Khambalia et al. [13] conducted a systematic review of the literature, identifying 50 studies published in English on the topic of anemia in indigenous peoples from 13 countries (Australia, Brazil, Canada, Guatemala, India, Kenya, Malaysia, Mexico, New Zealand, Sri Lanka, Tanzania, United States, and Venezuela). According to the authors, “*the burden of anemia is overwhelmingly higher among indigenous groups compared to the general population and represents a moderate (20–39.9%) to severe (≥ 40%) public health problem*”. Also according to these authors, anemia in indigenous children is most often preventable, being associated with the promotion of food security, improved living conditions and sanitation, and the treatment and prevention of parasitic diseases such as malaria and intestinal parasites.

Presently, about 300 indigenous ethnic groups, speakers of approximately 200 distinct languages, are identified in Brazil, constituting one of the national indigenous populations with the greatest ethnic diversity in the world [23,24]. Because these societies are socioculturally distinct from one another and from the national population, with heterogeneous forms of livelihood and social organization, the theoretical models of determination of diseases, including anemia, should be applied and interpreted with caution. Models such as those used in this paper are based in part on a set of variables, such as income and education, among others, which were originally derived from studies conducted in non-indigenous societies. Nevertheless, the use of such models for indigenous peoples in Brazil may be justified insofar as they increasingly participate in local and global markets, which in turn has important ramifications for their health, local economies, political systems, and social organizations, as is also occurring in other parts of the world [25,26]. Despite these caveats, the outcomes in the final models for anemia, which are discussed below, were associated with evaluated variables at all levels, as they are in many other studies on the epidemiology of childhood anemia.

The observed association between child’s age and anemia has been reported in several other studies worldwide [27-29]. Children under two years of age experience high rates of growth, which increases the demand for micronutrients such as iron, folate, and vitamin B_12_. The introduction of foods with low iron levels during weaning and elevated frequencies of infectious and parasitic diseases among young children are also important factors in determining childhood anemia. However, the relationship between child’s sex and anemia is less consistent, with some studies indicating associations between these variables [30,31] and others not [32-34]. The results of the National Survey point to an association between sex and anemia in indigenous children, with greater risk among boys, although the difference was small.

With regard to socioeconomic characteristics, the results of the National Survey indicate that both maternal schooling and the household goods index, which are often considered linked to family income, were shown to be protective factors for the occurrence of anemia in indigenous children in Brazil. As is well documented elsewhere, such dimensions are intimately related to the care received by children, including nourishment and access to health services [35-38].

Concerning the physical and sanitation characteristics of households, several variables showed a protective effect against the occurrence of anemia, including type of flooring, type of roofing, and source of drinking water. The use of non-durable materials, generally of plant origin, in home construction is often interpreted in the international epidemiological literature as a reflection of unfavorable socioeconomic conditions. However, care must be taken in interpreting these results because the use of plant-based raw materials (e.g., wood or palm thatch) in indigenous communities in Brazil does not necessarily derive from monetary poverty and is often an essential component of traditional household architectural techniques, especially in rural areas [39]. Nevertheless, as previously indicated, indigenous peoples in Brazil are experiencing rapid environmental, sociocultural, and economic transformations with repercussions for the variables associated with anemia. Whereas in some parts of Brazil, especially the North region, traditional indigenous home construction techniques are widespread, in other parts of the country with longer and more widespread histories of economic development, industrialized materials often predominate [17]. This contrast suggests that at least in certain portions of Brazil, such as the South/Southeast and Northeast, the presence of traditional plant-based construction materials may indicate unfavorable socioeconomic conditions as is understood to be the case in other populations globally.

The findings of the National Survey indicate an unexpected association between anemia and domestic source of drinking water. Several studies point to the protective effect of piped water available for domestic consumption on the occurrence of anemia in Brazil and elsewhere [40,41]. However, the present study shows indigenous children living in households relying on drinking water from rivers and lakes to present lower rates of anemia than those in households with access to piped water. Potentially, these children may have greater access to nutrient-rich local foods than those living in household with piped water but relying on low-cost industrial foods. Analysis of this possibility is beyond the scope of the present paper and deserves further investigation.

Maternal anemia was consistently associated with the occurrence of child anemia. Mothers and children most often share a home environment, which involves mutual exposure to a common set of physical, socioeconomic, and dietary conditions. This association gains relevance when considering that the prevalence of anemia among indigenous women was 32.7%, which is also extremely high [42-45]. Maternal iron deficiency is associated with low birth weight and prematurity, and there is evidence that even children born with adequate weight have diminished iron reserves when their mothers are anemic [35,46,47].

Among the analyzed variables related to children, low height-for-age and low weight-for-age remained associated positively with anemia after controlling for other variables. The association between these anthropometric indices and anemia has also been observed in several other studies [41,48]. Nutritional status, as assessed by both anthropometry and hemoglobin levels, is affected by a common set of factors, including socioeconomic status, sanitation, infectious and parasitic diseases, and diet. With regard to diet, protein-energy malnutrition favors the development of anemia through a synergistic relationship. Moreover, low hemoglobin levels have been implicated in compromising linear growth [49,50]. It remains unclear why weight-for-height showed an inverse association in our sample. Other factors recognized as important in determining child anemia, such as gestational age at birth and birth order, were not investigated here due to the impossibility of obtaining reliable data from local indigenous health services and the limitations of our data, which were restricted to children < 5 years [51,52].

Some limitations to this study should be taken into account when interpreting its results. The cross-sectional nature of the research design does not allow for the establishment of causal relationships. Moreover, the absence of data on infant feeding precludes analysis of dietary sources of bioavailable iron, which is important for understanding the epidemiology of anemia in the study population. The use of capillary blood instead of venous samples can constitute a source of bias due to the possibility of hemoglobin dilution with extracellular fluid through manipulation of the subject’s finger at the moment the technician pricks the skin and collects the blood drop. Nevertheless, this technique offers numerous widely recognized practical advantages and does not compromise the quality of diagnosis at the population level [53,54]. Additionally, the National Survey methodology did not address the etiology of anemia. This last point deserves particular attention because appropriate population treatment measures and intervention vary according to the disease’s etiology.

Iron deficiency is generally assumed to be the major cause of anemia globally [1,3]. In Brazil, dietary supplementation with iron sulfate for children between 6 and 18 months of age is a major nutritional initiative of the Ministry of Health that was implemented based on the assumption that most cases of anemia derive from dietary iron deficiency [46]. However, besides iron deficiency, other factors that may cause or be associated with anemia include nutritional deficiencies involving other micronutrients (e.g., folate, vitamin A, and vitamin B_12_), infectious and parasitic diseases (e.g., diarrhea, malaria, and geohelminthosis), glucose-6-phosphate dehydrogenase (G6PD) deficiency, and genetically derived hemoglobinopathies [2,27,55]. Recent research in the western Amazonian region of Brazil indicate that about a third of anemia cases observed in a sample of non-indigenous children was not associated with iron deficiency [56]. Similarly, studies of children living in the South and Northeast regions of Brazil demonstrate that sizable rates of children suffer from anemia caused by other factors other than iron deficiency [57-59].

Given this context, deeper knowledge about the etiology of anemia in indigenous children in Brazil is essential to its proper treatment and prevention. At present, there are no studies on nutritional deficiencies among indigenous populations in Brazil that measured serum folate, vitamin B_12_, plasma ferritin, or other micronutrients associated with anemia. Moreover, it is known that helminthiasis and diarrhea are highly prevalent in indigenous children in all regions of the country [60-64] and that about 70% of Brazil’s indigenous population lives in regions with high risk for transmission of malaria, a parasitic disease that can be particularly acute during childhood and can cause severe anemia [65-67]. Iron sulfate supplementation or treatment with standard dosages has low efficacy where there are high levels of infectious and parasitic diseases with the potential to impact nutritional status and, more specifically, to interfere directly on hemoglobin concentrations [68-70].

In conclusion, the findings of the National Survey reported here show widespread occurrence of anemia among indigenous children in Brazil, with prevalence rates being substantially higher than those among non-indigenous children. The findings also point to regional differences in the prevalence of anemia among indigenous children, with the highest rate being observed in the North region of the country. Considering the serious consequences of anemia for child health and development, as well as the preventable nature of iron-deficiency anemia, it is necessary to develop control and prevention strategies that attend to the sociocultural and nutritional specificities of anemia in indigenous children in Brazil.

## Abbreviations

CI: Confidence interval; CONEP: National Committee on Research Ethics (Comissão Nacional de Ética em Pesquisa); FUNASA: National Health Foundation (Fundação Nacional de Saúde); G6PD: Glucose-6-phosphate dehydrogenase; PNDS: National Survey on Demography and Health of Women and Children (Pesquisa Nacional de Demografia e Saúde da Criança e da Mulher); PR: Prevalence ratio.

## Competing interests

The authors declare that they have no competing interests.

## Authors’ contributions

AMC, CEAC, JRW, BLH, and RVS participated in the conception of the study. MSL, AMC, CEAC, JRW, PCIL, RVS, SAG, and ALE participated in data collection. MSL, AMC, ALE, BLH, and RVS performed data analysis. MSL, AMC, CEAC, JRW, and RVS contributed to the interpretation of data and wrote the paper. All authors participated in the revision of the manuscript and approved the version submitted for publication.
